# 

**Published:** 1878-10

**Authors:** 


### THE BUFFALO MEDICAL AND SURGICAL JOURNAL.

VOL. XVIII. OCTOBER, 1878. NO. 3.

